# Awareness and Uptake of Family Screening in Patients Diagnosed with Colorectal Cancer at a Young Age

**DOI:** 10.1155/2015/194931

**Published:** 2015-01-22

**Authors:** Niamh M. Hogan, Marion Hanley, Aisling M. Hogan, Margaret Sheehan, Oliver J. McAnena, Mark P. Regan, Michael J. Kerin, Myles R. Joyce

**Affiliations:** ^1^Department of Colorectal Surgery, University College Hospital Galway, Galway, Ireland; ^2^Discipline of Surgery, National University of Ireland, Galway, Ireland; ^3^Department of Histopathology, University College Hospital Galway, Galway, Ireland

## Abstract

*Background*. One-fifth of people who develop colorectal cancer (CRC) have a first-degree relative (FDR) also affected. There is a large disparity in guidelines for screening of relatives of patients with CRC. Herein we address awareness and uptake of family screening amongst patients diagnosed with CRC under age 60 and compare guidelines for screening. *Study Design*. Patients under age 60 who received surgical management for CRC between June 2009 and May 2012 were identified using pathology records and theatre logbooks. A telephone questionnaire was carried out to investigate family history and screening uptake among FDRs. *Results*. Of 317 patients surgically managed for CRC over the study period, 65 were under age 60 at diagnosis (8 deceased). The mean age was 51 (30–59). 66% had node positive disease. 25% had a family history of colorectal cancer in a FDR. While American and Canadian guidelines identified 100% of these patients as requiring screening, British guidelines advocated screening for only 40%. Of 324 FDRs, only 40.9% had been screened as a result of patient's diagnosis. *Conclusions*. Uptake of screening in FDRs of young patients with CRC is low. Increased education and uniformity of guidelines may improve screening uptake in this high-risk population.

## 1. Introduction

Recent reports estimate that colorectal cancer is responsible for 8% of all cancer deaths worldwide [1]. The major cause of death is development of distant metastasis in liver and lung, for which there are limited therapeutic options with curative intent [2]. Therefore, early detection is crucial as prognosis is heavily related to stage at diagnosis [2]. The association between family history and risk of developing colorectal cancer is well established [3]. For decades, it has been reported in the literature that familial colorectal cancer risk exists even in the absence of genetic heritability syndromes such as familial adenomatous polyposis [4, 5]. The risk of colorectal cancer in a patient with a positive family history in a first degree relative (FDR) has been estimated to be between 1.6- and 8-fold, with greatest risk when a relative has been diagnosed at a young age or with more than one affected FDR [5]. Despite this, there is a large disparity between screening recommendations in international guidelines with resultant physician uncertainty regarding requirement which patients should be screened and when. The present study assessed currently available screening guidelines by applying them to a cohort of patients diagnosed with colorectal cancer under the age of sixty. Awareness and uptake of screening amongst first degree relatives of patients diagnosed with colorectal cancer under the age of 60 are also assessed.

## 2. Methods

Institutional review board permission was sought and granted. The present study was conducted at a single centre, a tertiary referral centre for colorectal cancers in the west of Ireland. All patients with histologically confirmed colorectal cancer between June 2009 and May 2012 were identified from histopathology records. Patients under 60 years old were contacted. Those who agreed to participate were asked to complete a telephone questionnaire with detailed information regarding family history, awareness of risk, and uptake in screening amongst family members. Questions were also posed to establish if first degree relatives received or were offered a screening test as a result of the affected relative's diagnosis of colorectal cancer. International guidelines relating to screening family members of patients diagnosed with colorectal cancer at a young age were identified by literature review (British Society of Gastroenterology [6], American Society of Gastroenterology [7], and Canadian Society of Gastroenterology [8]). These were applied to the study cohort in order to identify the proportion of patients that would have been screened in accordance with different guidelines.

## 3. Results

There is significant variation in recommendations between international guidelines (Table 1).

**Figure 1:**
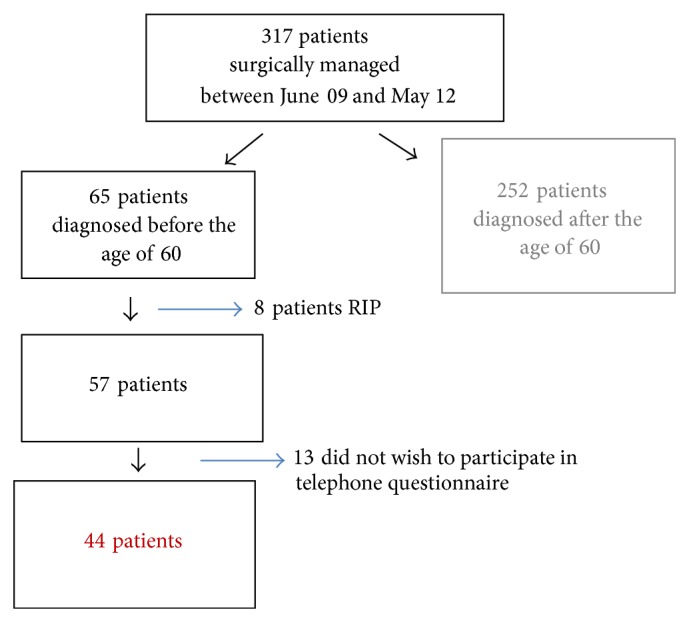
Study Cohort.

**Figure 2:**
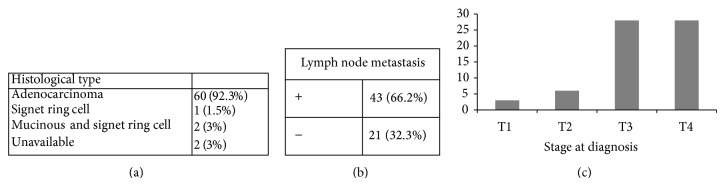
Tumour Characteristics: (a) tumour type, (b) nodal status, and (c) stage at diagnosis.

317 patients with pathologically confirmed colorectal cancer were identified during the study period (June 2009 to May 2012). 65 were under the age of 60. Eight of these patients were deceased at the time of conducting the questionnaire (12%) (Figure 1) (6 cancer related deaths, 2 unrelated). The demographic distribution of this cohort is outlined in Table 2. There was a slight male preponderance (56% male, 44% female). Mean age at diagnosis was 51 (30–59). The most common tumour type was adenocarcinoma (92%) (Figure 2). At time of diagnosis, 86% (*n* = 56) of patients had T3 or T4 disease. Mean lymph node yield was 15.3 (range 5–39) while 66% were lymph node positive.

Among the 57 living patients, there was an uptake of 79% (45 of 57 patients). Four patients refused participation, 1 patient was in a hospice, and 8 were not contactable by telephone. Of those who participated, 17 patients (38.6%) reported a family history of colorectal cancer with 11 patients reporting a history of colorectal cancer in a first degree relative (25%). 84% of patients recalled being asked about family history during their inpatient stay. 40% of patients were aware of a first degree relative who was screened as a result of their diagnosis. Of 324 living first degree relatives identified though thorough family history enquiries, 27.2% had been screened appropriately according to American and Canadian guidelines and 46.2% had been screened appropriately according to British guidelines. Anecdotally, reasons for lack of uptake were fear of cancer diagnosis and unwillingness to undergo colonoscopy.

A detailed family history was obtained from participants. The purpose of this focussed history was to assess how many, if any, cases of colorectal cancer had been diagnosed in first and second degree relatives of participants. This information was then used to assess which of this group, who all went on to develop colorectal cancer under the age of sixty, would have been offered screening based on diverse guidelines. To this end, British, American, and Canadian guidelines were then applied to the study cohort. While American and Canadian guidelines successfully identified 100% of these patients, British guidelines, which use a cut-off of family history under the age of 60 years, advocated screening for less than 40% of our study cohort.

## 4. Discussion

In contrast to the overall decreasing trend in incidence, in the United States rates of colorectal cancer are on the rise in younger patients [9]. Early detection of cancer vastly improves oncologic outcome [10]. This study cohort exhibits an aggressive disease phenotype. Of note, only patients who were surgically managed were included herein, which the authors recognize as a potential weakness of the present study. Significant debate exists in the literature regarding prognosis of colon cancer at a young age [11]. It has been established that patients diagnosed at a younger age tend to have tumours which are more aggressive, present at a later stage, and are associated with poorer pathologic findings [12]. Family history is known to be a prominent risk factor [13]. Early detection is crucial as young patients with early stage lesions have better overall 5-year survival rates than older counterparts [12]. In the series herein, the advanced disease phenotype at presentation highlights the importance of screening of first degree relatives of patients diagnosed with colorectal cancer at a young age. Despite this, significant variation in guidelines exists. In 2010, the British Society of Gastroenterology (BSG) and the Association of Coloproctology for Great Britain and Ireland (ACPGBI) published updated guidelines on screening for patients at moderate to high risk of colorectal cancer [6]. Adherence to guidelines, in the Irish context, is inconsistent. The Canadian Association of Gastroenterology and the Canadian Digestive Health Foundation guidelines published in 2010 [8] are largely based upon the American College of Gastroenterology guidelines published in 2008 [7]. While the American and Canadian guidelines performed well in recommending screening for all of the patients in our colorectal cancer cohort with FDR family history, the British guidelines would only have recommended screening for 36%. The remainder represent a group with a family member who was diagnosed at a young age with colorectal cancer and who themselves went on to develop the disease. Failure to recommend screening for these patients represents a missed opportunity since many patients presented with advanced disease. The main discrepancy between guidelines is the age at which screening is recommended. British guidelines suggest that those with only one affected first degree relative less than 50 years of age or two affected first degree relatives aged 60 years or older should undergo a once-only colonoscopy at age 55 years. The American College of Gastroenterology (ACG) guidelines recommend colonoscopy every five years beginning at age 40 years or 10 years younger than age at diagnosis of the youngest affected relative in single first degree relatives with colorectal cancer or advanced adenoma [7, 14].

## 5. Conclusion

A diagnosis of colorectal cancer at a young age represents a challenging scenario for patient and surgeon. Significant confusion exists regarding which family members should be screened and at what age. The findings of this study are likely to reflect the situation in the majority of centres in the UK and Ireland. Only a small number of dedicated family history clinics exist. Education of patients and their families regarding risk is minimal and this is perpetuated by a large disparity in recommendations of international guidelines. A recent study has concluded that the use of a population-based cancer registry to access the target population may have significant advantages in increasing uptake of screening in first degree relatives of those diagnosed with colorectal cancer [15]. Considering the advanced stage of disease at presentation exhibited by patients diagnosed with colorectal cancer under the age of 60, there is an urgent need for uniformity of international guidelines and a structured approach to deal with relatives in this high risk group.

## Figures and Tables

**Table 1:** International guidelines.

Guidelines for screening relatives		
(i) **British Society of Gastroenterology**	(i) **American College of Gastroenterology**	(i) **Canadian Association of Gastroenterology and the Canadian Digestive Health Foundation**
(ii) 2010	(ii) 2008	(ii) 2010

(i) Colorectal cancer in 1 FDR <50 years	(i) Colorectal cancer <60 years or advanced adenoma at any age in 1 FDR	(i) Colorectal cancer or adenomatous polyp <60 years in 1 FDR

(i) Once-only colonoscopy at age 55 years	(i) Colonoscopy at age 40 or 10 years younger than age of diagnosis of the youngest affected relative, whichever is first	(i) Colonoscopy at age 40 or 10 years earlier than the youngest diagnosis of polyp or cancer in the family, whichever comes first
(ii) If normal, no follow up.	(ii) Every 5 years	(ii) Every 5 years

**Table 2:** Demographic details.

Characteristic/demographic	Value, *n*
Age of diagnosis (years), mean, median (range)	51, 54, (30–59)
Age (years) by groups	
30–39	7 (10.8%)
40–49	13 (20%)
50–59	45 (69.2%)
Gender	
Male	36 (55.4%)
Female	29 (44.6%)
